# Spatiotemporal Alterations in Primary Odorant Representations in Olfactory Marker Protein Knockout Mice

**DOI:** 10.1371/journal.pone.0061431

**Published:** 2013-04-22

**Authors:** Marley D. Kass, Andrew H. Moberly, John P. McGann

**Affiliations:** Psychology Department, Behavioral and Systems Neuroscience Section, Rutgers, The State University of New Jersey, Piscataway, New Jersey, United States of America; Duke University, United States of America

## Abstract

Olfactory marker protein (OMP) is highly and selectively expressed in primary olfactory sensory neurons (OSNs) across species, but its physiological function remains unclear. Previous studies in the olfactory epithelium suggest that it accelerates the neural response to odorants and may modulate the odorant-selectivity of OSNs. Here we used a line of gene-targeted mice that express the fluorescent exocytosis indicator synaptopHluorin in place of OMP to compare spatiotemporal patterns of odorant-evoked neurotransmitter release from OSNs in adult mice that were heterozygous for OMP or OMP-null. We found that these patterns, which constitute the primary neural representation of each odorant, developed more slowly during the odorant presentation in OMP knockout mice but eventually reached the same magnitude as in heterozygous mice. In the olfactory bulb, each glomerulus receives synaptic input from a subpopulation of OSNs that all express the same odor receptor and thus typically respond to a specific subset of odorants. We observed that in OMP knockout mice, OSNs innervating a given glomerulus typically responded to a broader range of odorants than in OMP heterozygous mice and thus each odorant evoked synaptic input to a larger number of glomeruli. In an olfactory habituation task, OMP knockout mice behaved differently than wild-type mice, exhibiting a delay in their onset to investigate an odor stimulus during its first presentation and less habituation to that stimulus over repeated presentations. These results suggest that the actions of OMP in olfactory transduction carry through to the primary sensory representations of olfactory stimuli in adult mice *in vivo*.

## Introduction

Olfactory marker protein (OMP) is a 19 kDa intracellular protein expressed at high levels in olfactory sensory neurons (OSNs) in many vertebrate species [1], [2] with very limited expression elsewhere in the nervous system [3]. The molecular genetics [4], [5] and structure [6], [7], [8] of OMP are now well understood. OMP is expressed only late in cellular development and is widely used as a marker of mature OSNs [9], [10]. Perhaps in concert with this expression pattern, OMP may also play a role in epithelial neurogenesis [11] and axon guidance (possibly indirectly through changes in neuronal activity; see [12]).

The physiological role of OMP, an olfactory transduction protein, is increasingly well understood. Deletion of OMP in knockout mice produces smaller and slower OSN responses to odorants when recorded via electroolfactogram [13], slows odorant-evoked responses and reduces sensitivity in individual OSNs [14], and also causes significant perceptual changes, including elevated odor response thresholds [15]. No overall odor discrimination deficit has been observed in psychophysical tasks [16], but OMP-knockout mice do fail to exhibit a preference for their own mother over another lactating female [14]. Some of these neurophysiological and perceptual deficits can be rescued by viral vector-mediated gene replacement [17], [18], proving that these effects are directly caused by the lack of OMP. OMP's action is early in the olfactory transduction cascade, upstream of both cAMP and chloride channel activation [19], and it can modulate calcium extrusion from OSNs by the sodium/calcium exchanger [20]. Despite this body of research, the precise function of OMP in olfactory physiology remains unknown.

Interestingly, OMP-knockout mice exhibit changes in the spatiotemporal pattern of odorant-evoked epithelial voltage responses *in vitro* and presumably corresponding changes in perceived odor quality [16], [21]. In identified OSNs expressing the MOR23 receptor, immature (pre-OMP expression) OSNs and mature OSNs from OMP-knockout mice exhibited both slower responses to odorant stimulation and reduced sensitivity to odorants compared to mature, OMP- and MOR23-expressing OSNs [14]. Remarkably, this study also demonstrated an increase in odorant selectivity with OMP expression, such that OMP-null OSNs responded to a broader range of odorants than mature, OMP-expressing OSNs expressing the same receptor. However, it remains unclear how these differences in OSN response dynamics in the epithelium translate into corresponding alterations of OSN synaptic signaling to the brain. In the present study we compare spatiotemporal patterns of odorant-evoked OSN synaptic output *in vivo* and odorant-guided behavior in OMP-null and OMP-expressing mice to investigate the functional role of OMP in the olfactory system. Because these patterns indicate the output of the first neurons in the circuit, they are considered the primary neural representations of olfactory stimuli.

## Materials and Methods

### Ethics Statement

All experiments were performed in accordance with protocols approved by the Rutgers University Institutional Animal Care and Use Committee (Protocol Number 09-022). All surgeries were performed under anesthesia to minimize suffering.

### Subjects

Fifteen mice were used as subjects in the imaging experiments. All subjects were adult (8–9 months) females, and were gene-targeted mice expressing the synaptopHluorin (spH) exocytosis indicator [22] under control of the OMP promoter, replacing the OMP coding region as previously reported [23]. In these mice, spH is expressed in mature OSNs, and odorant-evoked spH signals linearly indicate neurotransmitter release from OSN terminals into olfactory bulb glomeruli [23], [24]. Homozygous and heterozygous OMP-spH mice were generated as described in [25]. Briefly, the homozygous OMP-spH mice were from a previously reported line with an albino C57BL/6 background (provided by Dr. Matt Wachowiak; see [25]). These mice are OMP-null (OMP^−/−^) because they have had both copies of the OMP coding region replaced with spH. This OMP^−/−^ line was crossed with a wild-type 129SvJ line to produce F1 hybrid offspring. The progeny from this cross were thus heterozygous for spH and OMP (OMP^−/+^) and on a mixed C57BL/6 and 129 background, analogous to those used by [14].

To compare the odorant-guided behavior of OMP^−/−^ and wild-type mice on the same background, we compared the investigation behavior of 11 OMP^−/−^ mice (as described above; see [25], [26]) with that of 19 wild-type (non-albino) C57BL/6 mice obtained from Charles River Laboratories (strain code #027). Subjects used for behavioral experimentation were adults of mixed sexes.

### Surgical Procedures

Bilateral cranial windows were surgically implanted, as previously reported [27], [28]. Briefly, mice were anesthetized via intraperitoneal administration of a 10 mg/mL pentobarbital (Sigma-Aldrich, St. Louis, MO) solution at a volume of 1 mL/0.01 kg, and additional boosters were administered as needed to maintain deep anesthesia throughout the duration of the imaging preparation. Between 0.3 and 0.5 mL of 0.25% bupivacaine HCl (Sigma-Aldrich, St. Louis, MO) was administered subcutaneously along the scalp to provide local anesthesia (varied based on animal size). Mucous secretions were reduced via subcutaneous administration of 1 mL/0.01 kg of 0.1% atropine sulfate (Sigma-Aldrich, St. Louis, MO). During deep anesthesia, body temperature was monitored via rectal probe thermometry and was maintained at 38±0.5°C via a feedback-regulated heating pad (TC-1000, CWE Inc, Ardmore, PA).

The scalp was shaved, wiped down with a topical antiseptic bactericide, and then surgically removed. The periosteal membrane was removed and the skull was cleaned and dried with a 70% ethanol solution. The skull was fitted with a custom-sized dental acrylic head cap that was bolted into a head bar which was then mounted onto a head holder. The bone overlying the dorsal surface of both olfactory bulbs was thinned with a hand-held dental drill. Ringer's solution, containing 140 mM NaCl, 5 mM KCl, 1 mM CaCl_2_, 1 mM MgCl_2_, 10 mM HEPES, and 10 mM dextrose, was applied to the surface to turn the thinned bone transparent. The window was finally topped with a glass cover slip and the preparation was immediately transferred from the stereomicroscope (Leica MZ12) to the imaging apparatus for *in vivo* optical imaging under anesthesia.

### *In Vivo* Visualization of Odorant-Evoked spH Signals

Odorant-evoked OSN synaptic output into glomeruli on the dorsal surface of the olfactory bulbs was imaged *in vivo*, as previously described [25], [27], [29]. Briefly, a custom imaging apparatus was used to record spH signals *in vivo*. Fluorescence epi-illumination was used on an Olympus BX51 microscope with a 4× objective and illumination was provided by a 470 nm wavelength bright light-emitting diode (Thorlabs, Newton, NJ). Light levels were adjusted to approximately standardize baseline fluorescence levels across mice, which helps to control for any differences in optical clarity and spH expression. A filter set containing HQ480/40 excitation, Q505LP dichroic, and HQ535/50 emission filters was used to visualize spH signals. A monochrome CCD camera (NeuroCCD, SM-256, RedShrtImaging, Decatur, GA) was used for image acquisition. Optical signals were acquired at a pixel resolution of 256×256 and at a frame rate of 7 Hz.

A panel of 4 odorants that are known to activate glomeruli on the dorsal surface of the olfactory bulbs, including *n*-butyl acetate (BA), methyl valerate (MV), 2-hexanone (2HEX), and *trans*-2-methyl-2-butenal (2M2B) (at 95–99% purity, Sigma-Aldrich, St. Louis, MO), was presented via an 8 channel, air dilution olfactometer using nitrogen as the carrier, as previously described [25], [27], [29]. Cross-contamination between odorants was prevented by using dedicated lines for each odor. A mass flow controller (Aalborg, Orangeburg, NY) operating through software written for MatLab (Mathworks, Natick, MA) was used to perform odorant dilution. Concentration is expressed in arbitrary units (a.u.) and all 4 odorants in the panel were presented at 3 target concentrations: 7.5 a.u., 15 a.u., and 30 a.u. Note that prior to each day's experiments the olfactometer was calibrated to ensure that all subjects were presented with the same target odorant concentrations during imaging. To perform this calibration, the complete odor panel was tested by taking multiple measurements of each of the 12 stimuli with a photoionization detector (ppbRAE3000, RAE Systems, CA) and then adjusting the odorant dilution as needed to achieve the desired target concentrations. This method yielded precisely quantified stimulus concentrations that were reliably reproduced across preparations.

The odorant delivery manifold was positioned approximately 2 cm in front of the mouse's nose. Each concentration of each odorant was delivered in a block of 4 trials that were presented with 60 sec inter-trial intervals (ITIs). Individual trials were 16 sec in duration and consisted of a 4 sec pre-odor baseline, 6 sec odor presentation, and 6 sec post-odor recovery period. Two blocks of blank (no odor) trials, each containing 6, 16 sec blank trials that were presented at 60 sec ITIs, were taken (1 block at the beginning and 1 block at the end of the preparation) and then averaged together. The blank (no odor) average was then subtracted from each odorant trial to correct for photobleaching. The 4 blank-subtracted trials in each odor block were averaged together to improve the signal-to-noise ratio of the data and generate an overall response map for each concentration of each odor.

**Figure 1:**
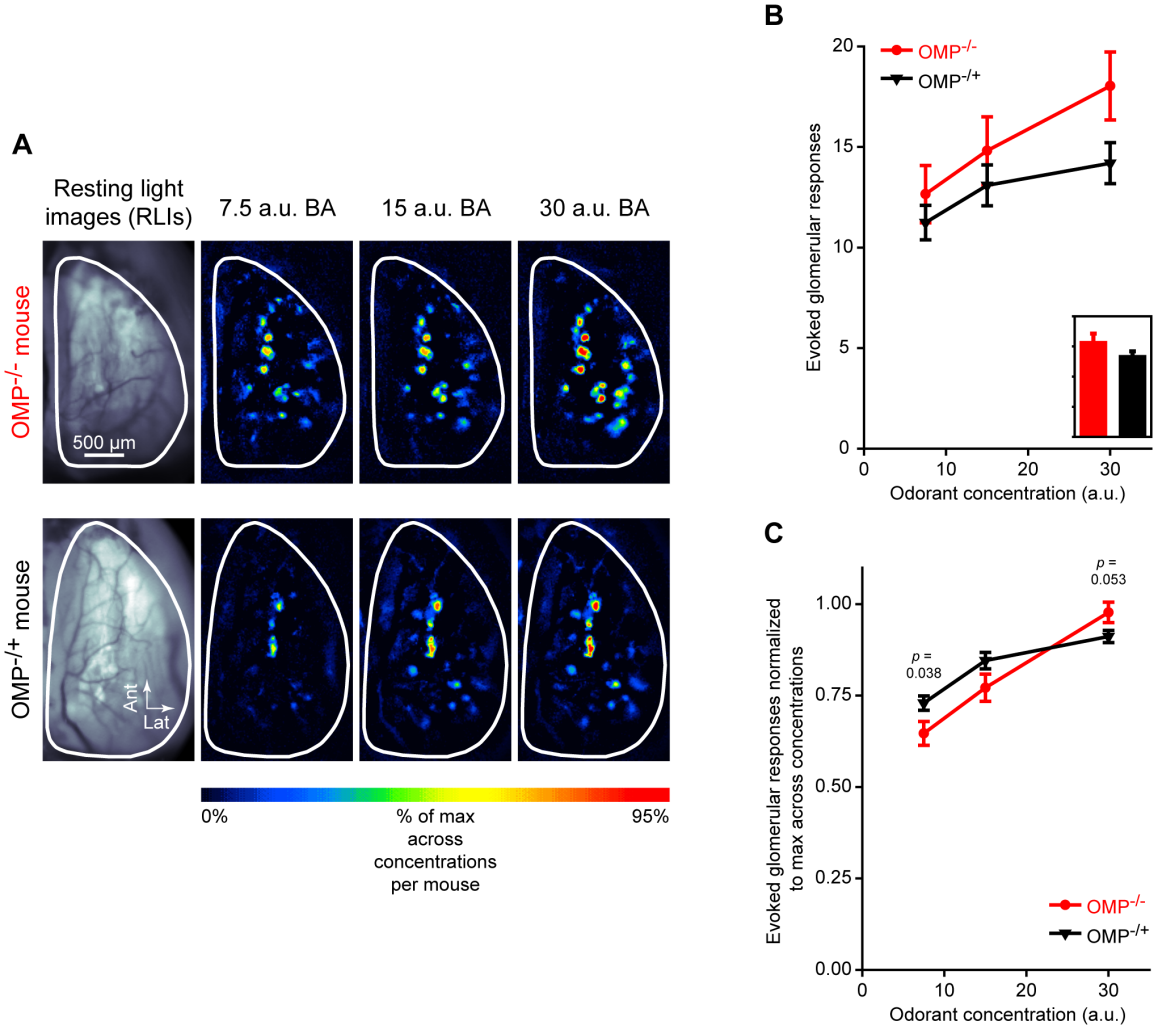
The number of glomeruli receiving odorant-evoked OSN input is enhanced in OMP^−/−^ mice. (**A**) Resting light images (RLIs) of the dorsal surface of the olfactory bulb through the cranial window (*first column*) and pseudocolored response maps evoked by 3 concentrations of butyl acetate (BA) (*second-fourth columns*). The *top row* is an example from an OMP^−/−^ mouse and the *bottom row* is an example from an OMP^−/+^ mouse. (**B**) Mean (±SEM) number of evoked glomerular responses plotted as a function of odorant concentration. The overall group means shown in the *inset*, which is scaled to the *y*-axis of **B**, are averaged across all odors and concentrations. (**C**) Mean (±SEM) number of glomerular responses normalized to the maximum number of evoked responses across 3 odorant concentrations. In **B** and **C** OMP^−/−^ mice and OMP^−/+^ mice are shown in *red* and *black*, respectively.

**Figure 2:**
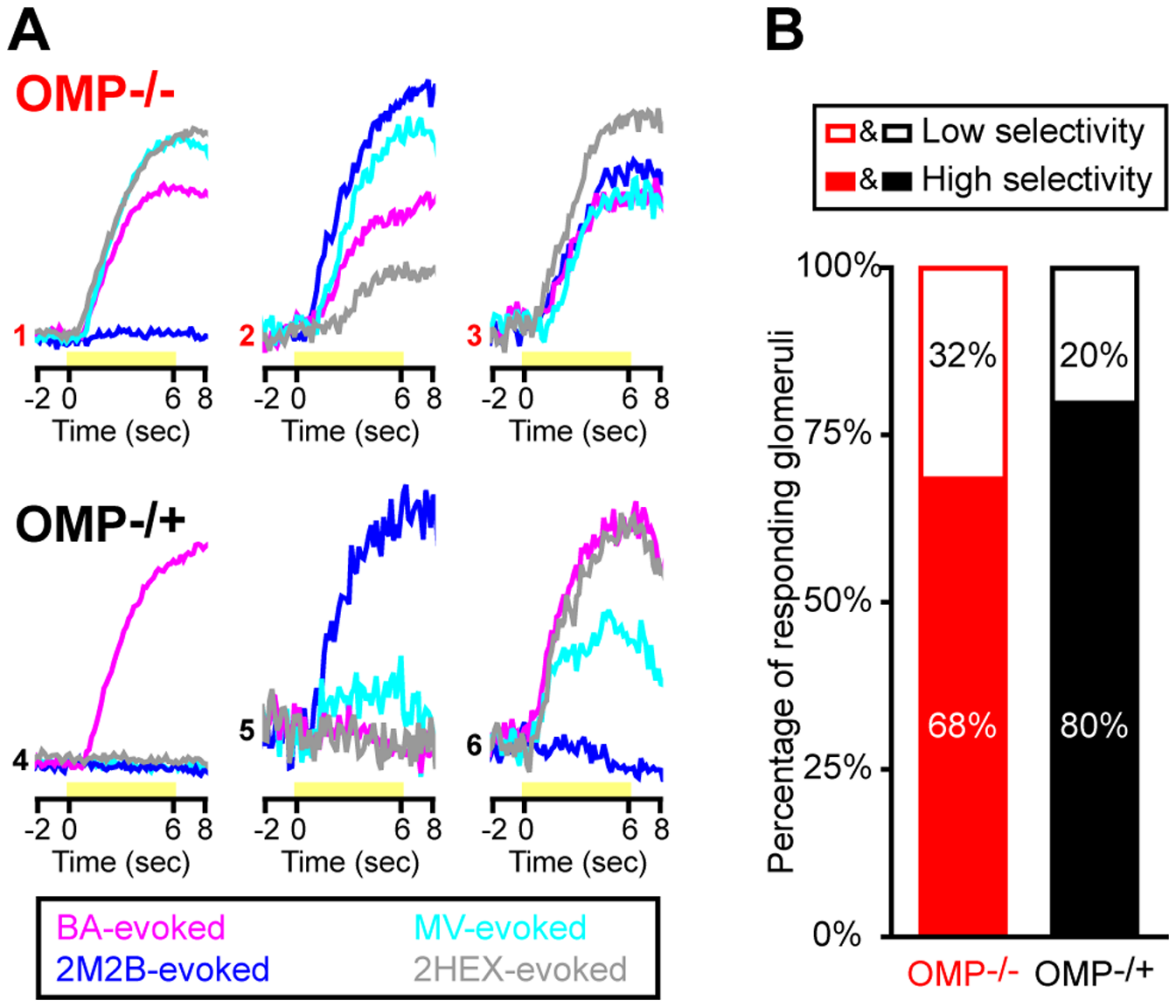
Odorant response selectivity is reduced in glomeruli from OMP^−/−^ mice. (**A**) Example odorant response selectivity patterns in 6 individual glomeruli; 3 OMP^−/−^ glomeruli (*top row*, traces 1–3) and 3 OMP^−/+^ glomeruli (*bottom row*, traces 4–6). Each set of 4 traces corresponds to a single glomerulus' responsivity to 4 test odorants that were all presented at a concentration of 30 a.u.: butyl acetate (*BA, magenta*), methyl valerate (*MV, cyan*), *trans*-2-methyl-2-butenal (*2M2B, blue*), and 2-hexanone (*2HEX, grey*). Each set of traces is scaled relative to the maximum evoked response across all 4 odorants per glomerulus. Yellow bars indicate the time when odor stimuli were presented. (**B**) Percentage of OMP^−/−^ (*red*) and OMP^−/+^ (*black*) glomerular populations that were categorized as having higher (*closed bars*) or lower (*open bars*) odorant response selectivity.

**Figure 3:**
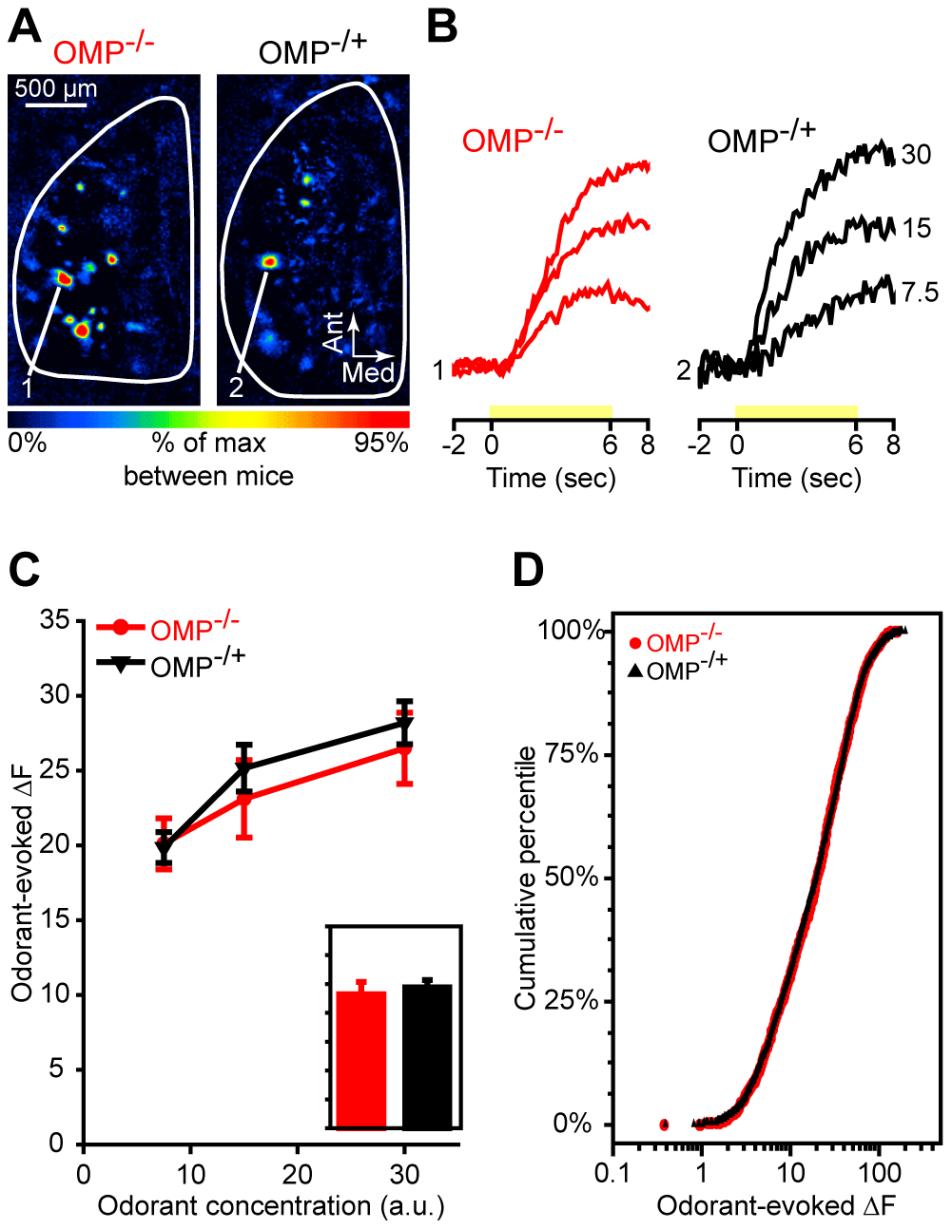
The magnitude of peak spH responses is unaltered in OMP^−/−^ mice. (**A**) Pseudocolored difference maps from an OMP^−/−^ mouse (*left*) and an OMP^−/+^ mouse (*right*). These maps were evoked by presentation of 2HEX at a concentration of 30 a.u. (**B**) Sets of traces from an OMP^−/−^ mouse (*left*) and an OMP^−/+^ mouse (*right*) corresponding to the numbered callouts in **A**. Traces were evoked by 3 concentrations of 2HEX (labeled on the right in a.u.) and are all scaled relative to the maximum response across concentrations and between the two glomeruli. Yellow bars indicate time of 2HEX presentation. (**C**) Mean (±SEM) odorant-evoked change in fluorescence (ΔF) plotted as a function of odorant concentration. The overall group means shown in the *inset*, which is scaled to the *y*-axis of **C**, are averaged across all odors and concentrations. (**D**) Cumulative probability plot showing the distributions of odorant-evoked ΔF values for populations of glomerular responses in OMP^−/−^ mice and OMP^−/+^ mice. ΔF distributions for each group are pooled across 3 concentrations of 4 test odorants. In **B**, **C**, and **D** OMP^−/−^ mice and OMP^−/+^ mice are shown in *red* and *black*, respectively.

**Figure 4:**
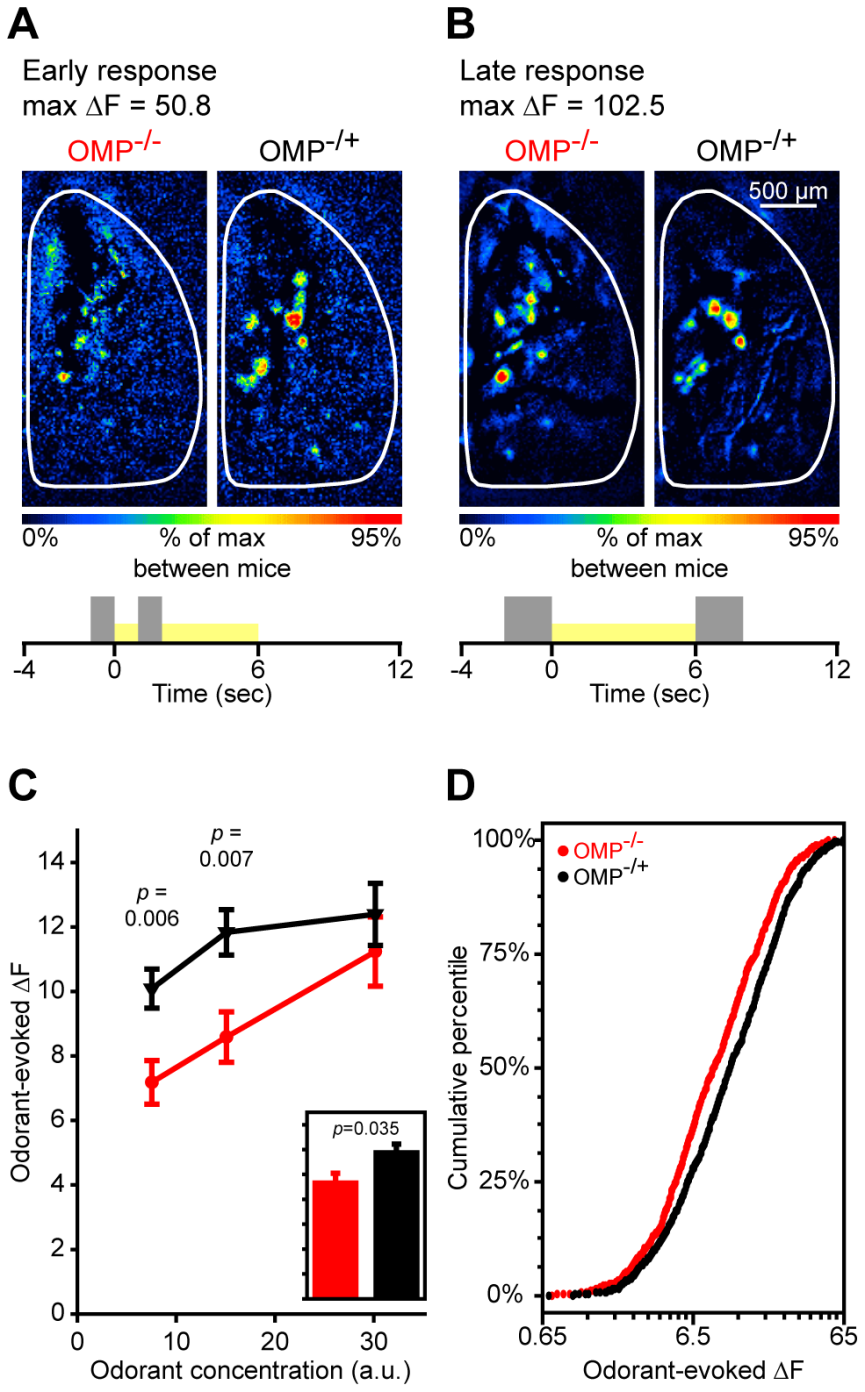
The evolution of odorant-evoked maps is slowed in OMP^−/−^ mice. (**A**) Pseudocolored difference maps from an OMP^−/−^ mouse (*left*) and an OMP^−/+^ mouse (*right*) showing an early response evoked by 7.5 a.u. MV. (**B**) Late phase of the same responses shown in **A**. Trial summaries shown at the bottom of **A** and **B** indicate the subtractions that were made to generate early and late response maps, respectively. (**C**) Mean (±SEM) odorant-evoked change in fluorescence (ΔF) during the early response phase (see subtraction shown in **A**) plotted as a function of odorant concentration. The overall group means shown in the *inset*, which is scaled to the *y*-axis of **C**, are averaged across all odors and concentrations. (**D**) Cumulative probability plot showing the distributions of early odorant-evoked ΔF values for populations of glomerular responses in OMP^−/−^ mice and OMP^−/+^ mice. ΔF distributions for each group are pooled across 3 concentrations of 4 test odorants. In **C** and **D** OMP^−/−^ mice and OMP^−/+^ mice are shown in *red* and *black*, respectively.

The fluorescence imaging data were quantified as previously reported [29]. Briefly, odorant-evoked spH signals for traces from each pixel overlying a responsive glomerulus were determined by subtracting the baseline fluorescence (defined as the average value of 15 frames collected immediately prior to odor onset) from the peak odorant-evoked change in fluorescence (ΔF, defined as the average value of 15 frames centered around the peak trace inflection after odor onset). Because spH provides an integrative signal of exocytosis over time, this peak typically occurred near the end of the odorant presentation and was considered a “late” response signal and used to evaluate overall differences between groups in odor maps (Figures 1, 2, and 3). To evaluate differences in odor maps that may occur earlier in the response a second subtraction was performed, whereby the average of 7 frames (1 sec) immediately prior to odor onset were subtracted from the average of 7 frames beginning 2 sec after odor onset (shown in Figure 4A). All data were high-pass filtered with a Gaussian spatial filter through software written for MatLab (Mathworks, Natick, MA). Responsive glomeruli were first hand-selected as regions of interest and then confirmed statistically; when a glomerulus' average response to an odorant was more than 3 standard errors (SEs) greater than 0, it was counted as a response.

### Imaging Data Analysis

All data were processed in Neuroplex and Matlab and then exported to SPSS 17.0 for statistical analysis and to SigmaPlot 11.0 and STATVIEW for graphing. Data used to perform the late response signal analyses (results shown in Figures 1, 2, and 3) came from 654 responsive glomeruli in 22 olfactory bulbs from 11 OMP^−/+^ mice and 249 responsive glomeruli in 8 olfactory bulbs from 4 OMP^−/−^ mice. The early response signal analyses (results shown in Figure 4) were performed on a subset of the data that was collected from 264 early-responsive glomeruli in 10 olfactory bulbs from 5 OMP^−/+^ mice and 190 early-responsive glomeruli in 8 olfactory bulbs from 4 OMP^−/−^ mice.

The number of evoked glomerular responses and the magnitude of those responses were first analyzed with omnibus mixed-model ANOVAs, with odorant (BA; MV; 2HEX; 2M2B) and concentration (7.5 a.u.; 15 a.u.; 30 a.u.) as within-subjects factors and OMP expression (OMP^−/−^; OMP^−/+^) as a between-subjects factor. These overall analyses were followed up with *post hoc* tests (including additional ANOVAs and *t* tests) to perform planned comparisons and further evaluate interactions of interest. To investigate odorant response selectivity each observed glomerular response was categorized based on 3 parameters: 1) OMP expression (OMP^−/−^ or OMP^−/+^), 2) odorant selectivity (highly selective, responding to only 1 or 2 odorants in the panel; non-selective, responding to 3 or 4 odorants in the panel), and 3) concentration sensitivity (responding to 1–2 or 3–4 odorants at 7.5 a.u., 15 a.u., or 30 a.u.). The cross-tabulated frequency data were then analyzed via log-linear regression to determine if the odor selectivity that we observed in responsive glomeruli could be best accounted for by a model including all 3 categorical variables. Additional χ^2^ tests were performed for *post hoc* comparisons. The α value for accepting a significant result in all tests was 0.05.

### Behavioral Testing and Analysis

**Figure 5:**
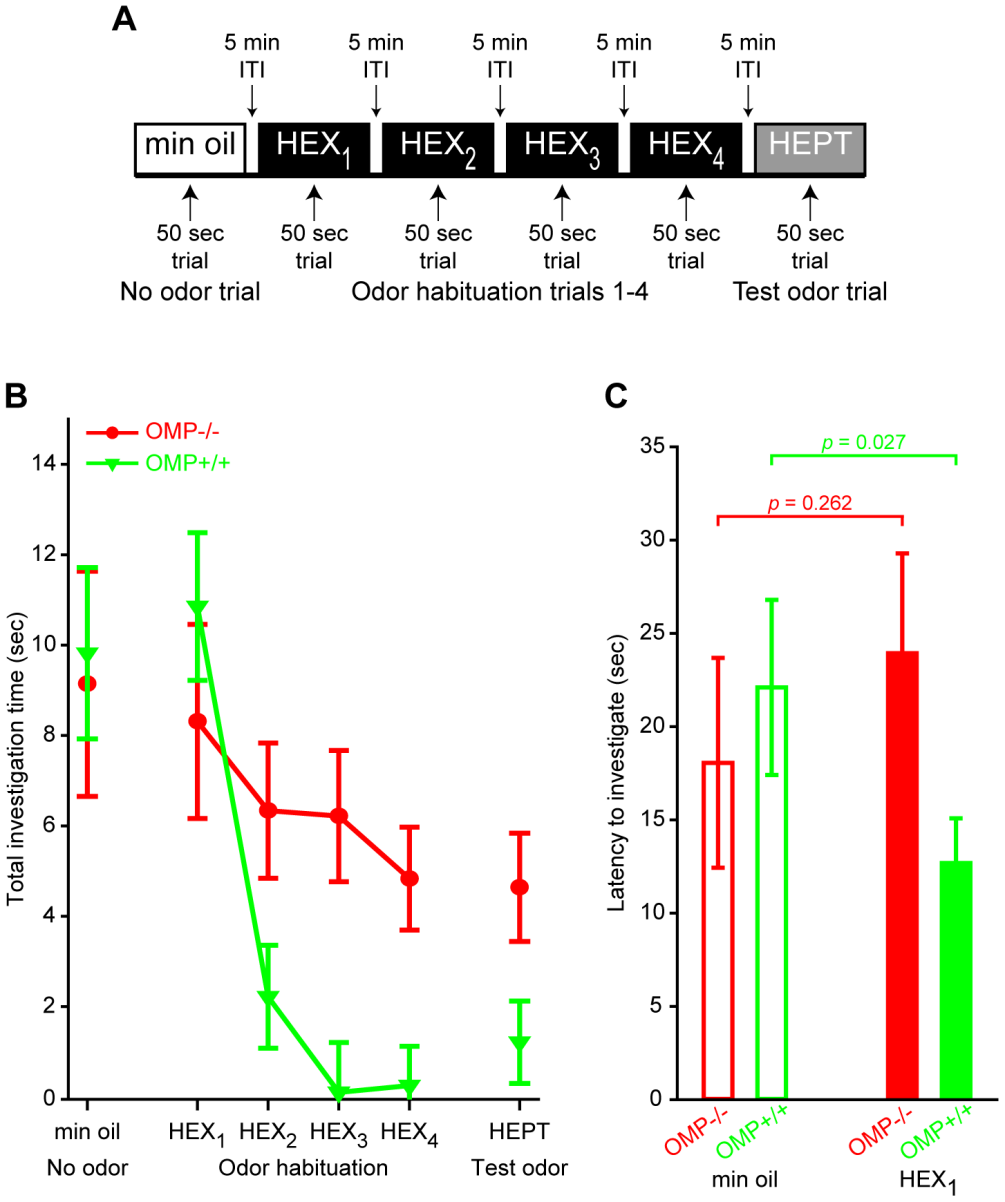
Non-associative odor-induced behaviors are altered in OMP^−/−^ mice. (**A**) Procedure summary of behavioral testing protocol. (**B**) Mean (±SEM) duration of stimulus investigation (sec) plotted as a function of trial type. (**C**) Mean (±SEM) latency (sec) to begin investigating the mineral oil (no odor) stimulus (*open bars*) and the HEX stimulus (*solid bars*) during the first and second trials, respectively, of the paradigm shown in **A**. In **B** and **C** OMP^−/−^ mice and OMP^+/+^ mice are shown in *red* and *green*, respectively.

Investigation of a novel odor stimulus and subsequent odor habituation and cross-habituation/dis-habituation was assessed in 29 mice that were single-housed in standard open shoebox cages (15 cm×12 cm×25 cm WxHxL). During testing, the home cage was transferred from the colony room to the behavioral testing room and all procedures were then carried out in the home cage. During each trial a hexagonal weigh boat (6.4 cm top; 4.7 cm base) containing filter paper treated with 0.6 mL of solution was placed on the wire cage top. As shown in the procedure summary in Figure 5A, the first trial of a behavioral testing session was a mineral oil only (no odor) trial that was used to acclimate subjects to the procedures and assess baseline levels of activity. The second through fifth trials were odor habituation trials, where the filter paper was treated with hexaldehyde (HEX, Sigma-Aldrich, St. Louis, MO), and the sixth trial was a test trial in which the odor was switched to filter paper treated with heptaldehyde (HEPT, Sigma-Aldrich, St. Louis, MO). Both odorants were diluted in mineral oil in proportion to their respective vapor pressures, thus producing equivalent vapor concentrations (HEX diluted to ∼0.01%; HEPT diluted to ∼0.26%). When measured with a photoionization detector, these odor stimuli were also approximately equivalent to the lowest concentration (i.e., 7.5 a.u.) at which each of the 4 imaging odorants were presented (see above). We selected HEX and HEPT as an odor pair for this behavioral assessment because the 2 odorants are chemically similar and potentially difficult to discriminate, which should facilitate the detection of small differences in the discrimination abilities of OMP^−/−^ and OMP^+/+^ mice. As shown in Figure 5A, all trials were 50 sec in duration and given at 5 min ITIs. The experimenter manually scored investigation time (in sec) in the “odor zone” (a clearly demarcated 15 cm×12 cm×10 cm WxHxL region underlying the stimulus) during all trials. All behavioral sessions were recorded so that a sample of trials could then be scored by a second experimenter to confirm inter-observer reliability.

To assess odor habituation, a non-associative form of learning, investigation during the second through fifth trials (i.e., HEX-presented trials 1–4) was analyzed with an overall OMP expression (OMP^−/−^; OMP^+/+^) × trial (HEX_1_; HEX_2_; HEX_3_; HEX_4_) mixed ANOVA. Planned *post hoc* ANOVAs and *t* tests were also used to evaluate habituation rates per group and group differences per trial. Detection of an odor stimulus was also assessed by comparing the total investigation time during the first (no odor) and second (HEX_1_) trials (see procedure summary in Figure 5A). The latency to begin stimulus investigation during these 2 trials was scored from video recordings of all OMP^−/−^ subjects and from 14 OMP^+/+^ subjects, and then used as an additional measure of initial odor detection abilities. Note that we expected the animals to reduce (or potentially stop) their investigation of the odor stimulus across trials. Consequently, we restricted our analysis on investigation latency to the mineral oil only trial and the HEX_1_ trial. Both behavioral parameters, which were 1) the total stimulus investigation time during each 50 sec trial and 2) the latency to begin stimulus investigation, were analyzed with 2-way ANOVAs and planned *post hoc t* tests. Cross-habituation/dis-habituation to a different, but highly similar test odorant was determined by comparing investigation time during the fourth odor habituation trial to investigation time during the test odor trial (Figure 5A & 5B; HEX_4_ versus HEPT).

## Results

### The Number of Odorant-Evoked Glomerular Responses Is Increased in OMP^−/−^ Mice

To determine if there were differences between OMP^−/−^ and OMP^−/+^ mice in the number of odorant-evoked glomerular responses, the number of responses evoked by a panel of 4 odorants presented at 3 concentrations each was quantified per olfactory bulb in each mouse and then analyzed via a factorial ANOVA, as described above. As shown in Figure 1A and 1B, the number of glomeruli receiving odorant-evoked nerve output tended to be greater in OMP^−/−^ mice than in OMP^−/+^ mice, and this increase became more robust as the concentration of the odor stimulus was increased (significant genotype × concentration interaction, *F*_(2, 56)_ = 6.455, *p* = 0.003, η_p_^2^ = 0.187). However, there was no main effect of OMP expression on the overall number of evoked responses (Figure 1B, inset; *F*_(1, 28)_ = 1.625, *p* = 0.213). This suggests that OMP may play a role in the rate of glomerular recruitment, a phenomenon that is observed when odor stimuli are presented at increasing concentrations [23], [30], [31].

To test this hypothesis we normalized the number of evoked glomerular responses at each concentration relative to the maximum number of evoked responses across the 3 odorant concentrations that were presented. This normalization was performed within each test odorant per olfactory bulb. The normalized values were then analyzed via a genotype (OMP^−/−^; OMP^−/+^) × odorant (BA; MV; 2HEX; 2M2B) × concentration (7.5 a.u.; 15 a.u.; 30 a.u.) mixed ANOVA. If OMP is not involved in concentration-dependent glomerular recruitment, then the normalized concentration response curve for OMP^−/−^ mice should be identical to that of OMP^−/+^ mice. However, if OMP does play a role in this phenomenon, then the normalized concentration response functions should differ, and the slope corresponding to the rate of glomerular recruitment in OMP^−/−^ mice should be steeper. Consistent with our hypothesis, this normalization identifies significantly differing rates of recruitment based on OMP expression (Figure 1C) including a significant genotype × concentration interaction (*F*_(2, 56)_ = 4.467, *p* = 0.016, η_p_^2^ = 0.138; *post hoc* group comparisons shown in Figure 1C). Specifically, the effect of concentration is greater in OMP^−/−^ mice (*post hoc* odorant × concentration ANOVA, *F*_(2, 14)_ = 49.854, *p*<0.001, η_p_^2^ = 0.877) than in OMP^−/+^ mice (*post hoc* odorant × concentration ANOVA, *F*_(2, 42)_ = 17.395, *p*<0.001, η_p_^2^ = 0.453) demonstrating that the number of evoked responses in OMP^−/+^ mice saturates more quickly than in OMP^−/−^ mice. These data thus indicate a role for OMP in normal glomerular recruitment.

### OSN Odorant Selectivity Is Decreased in OMP^−/−^ Mice

OMP is necessary for the development of mature odorant response specificity in OSNs [14]. Thus, it is possible that the increased number of glomerular responses evoked by the 4-odorant panel in OMP^−/−^ mice (see Figure 1B) is caused not by the integration of additional glomeruli in OMP^−/−^ mice but by each glomerulus responding to more than one odorant (i.e., a difference in odorant response selectivity). Accordingly, we tested the hypothesis that individual odorant-responsive glomeruli in OMP^−/−^ mice were more likely to respond to multiple odorants in our panel than those in OMP^−/+^ mice.

To test this we first quantified the number of odorants evoking a response in each individual glomerulus (ranging from 1–4). Next, we collapsed the glomeruli responding to 1 or 2 odorants into a single “higher selectivity” category and the glomeruli responding to 3 or 4 odorants into another “lower selectivity” category. For example, in Figure 2A the sets of traces corresponding to glomeruli 4 and 5 are categorized as having higher selectivity, whereas the sets of traces corresponding to glomeruli 1–3 and 6 are characterized as having lower selectivity. Note that collapsing into the two higher/lower categories greatly simplifies analysis and presentation, but a parallel analysis using all 4 original values produced similar results. As described above (see Materials and Methods), we then used log-linear regression to analyze glomerular response selectivity across odorant concentrations. Individual glomerular responses were categorized best by a model that included all 3 variables (i.e., genotype, odorant response selectivity, and concentration). Specifically, a larger percentage of the OMP^−/−^ glomerular population was categorized as having lower response selectivity (see sample traces 1–3 relative to sample traces 4–6 shown in Figure 2A) than that of the OMP^−/+^ glomerular population (partial χ^2^_(1)_ = 36.698, *p*<0.001, shown in Figure 2B). The difference in the selectivity of glomerular populations was also enhanced at higher odorant concentrations (3-way association, ÷^2^_(2)_ = 7.295, *p* = 0.026). For example, when the imaging odor panel was presented at a concentration of 30 a.u. (the highest concentration presented) 45% of OMP^−/−^ glomeruli were categorized as having low response selectivity, whereas only 26% of OMP^−/+^ glomeruli were placed in the same category.

### Total Odorant-Evoked Nerve Output Is Unaltered in OMP^−/−^ Mice

The magnitude of odorant-evoked OSN synaptic output into each responsive olfactory bulb glomerulus was calculated by subtracting the average of ∼2 sec of baseline frames from the average of ∼2 sec of frames collected at the peak of the spH signal, normally at or about the odor stimulus offset (see Figure 4B for details). This difference, representing the odorant-evoked change in fluorescence (ΔF, response magnitude), was quantified for each responding glomerulus. The average of all evoked glomerular responses was then separately calculated for the 4 odors (and 3 concentrations) presented to each mouse. The overall average response magnitudes were analyzed via mixed model ANOVA, as described above.

As shown in Figures 3A, 3B, and 3C (*inset*, *F*_(1, 28)_ = 0.224, *p* = 0.64, η_p_^2^ = 0.008), peak odorant-evoked ΔF did not differ between OMP-null and heterozygous mice, and this was true across all 3 odorant concentrations that were presented (Figure 2B & 2C; group × concentration interaction, *F*_(2, 56)_ = 1.179, *p* = 0.315, η_p_^2^ = 0.04). In addition to our parametric analysis using the averages (representing the overall magnitude of evoked responses, as described above), we assessed the distributions of response magnitudes (see Figure 3D). Individual ΔF values for glomeruli in OMP^−/−^ mice and OMP^−/+^ mice were ranked evenly (Mann-Whitney *U*, M-W *U*, *Z* = −0.732, *p* = 0.71), and the overall distributions of ΔF values in OMP^−/−^ and OMP^−/+^ glomerular populations were indistinguishable (Figure 3D; Kolmogorov-Smirnov, K-S, *Z* = 0.805, *p* = 0.535). These data demonstrate that the total synaptic output from OSNs evoked by a 6 sec odorant presentation is not reduced in OMP knockout mice.

### Odor Maps Develop on a Longer Time Scale in OMP^−/−^ Mice Than in OMP^−/+^ Mice

OSN response kinetics are slowed without OMP expression [13], [14]. Thus, while the peak amplitudes of the odorant-evoked spH signals do not differ between OMP^−/−^ mice and OMP^−/+^ mice (see Figure 3), it is possible that there may be a difference in the time course of these responses. To address this we generated early response maps from a subset of mice in both groups by subtracting the average of 1 sec of frames collected immediately prior to odor onset from the average of 1 sec of frames beginning 2 sec after odor onset (see Figure 4A). This allowed us to examine early differences in the evolution of odor maps with higher resolution (i.e., the initial subtraction is generated from 2 sec averages).

As shown in Figure 4A, the early MV-evoked response map in an OMP^−/−^ mouse (left) exhibits smaller responses and is less spatially distinct than that seen in an OMP^−/+^ mouse (right). However, no difference is observed between mice in response magnitudes at the late peak of the response in MV-evoked response maps in the same subjects (Figure 4B). Interestingly, there is a large difference between the early and late MV-evoked response magnitudes in the OMP^−/−^ subject (compare left maps in Figures 4A & 4B), but not in the OMP^−/+^ subject (compare right maps in Figures 4A & 4B). On average, the odorant-evoked ΔF values were significantly smaller in OMP^−/−^ mice than in OMP^−/+^ mice when measured early in the odorant presentation (overall main effect of group shown in Figure 4C, inset; *F*_(1, 16)_ = 5.287, *p* = 0.035, η_p_^2^ = 0.248). As indicated by the significant genotype × concentration interaction (means from 2-way interaction shown in Figure 4C; *F*_(2, 32)_ = 3.915, *p* = 0.03, η_p_^2^ = 0.197), this difference was most pronounced when odorants were presented at concentrations of 7.5 and 15 a.u., regardless of the imaging odorant stimulus (non-significant genotype × concentration × odorant interaction, *F*_(6, 96)_ = 0.195, *p* = 0.978, η_p_^2^ = 0.012). Additionally, when we took each individual response into account the ΔF values that were observed in OMP^−/−^ mice were ranked consistently lower than that in OMP^−/+^ mice (Figure 4D; M-W *U*, *Z* = −5.882, *p*<0.001), and the distributions overall differed as well (Figure 4D; K-S, *Z* = 2.696, *p*<0.001). These results are consistent with previous reports from the epithelium [13], [14], [17] and suggest that the development of primary representations of odor stimuli is slower in OMP^−/−^ mice.

### OMP^−/−^ Mice Exhibit Attenuated Odor Habituation and Delayed Odor Investigation

We hypothesized that if the altered physiological responses to odor stimuli (Figures 1, 2, and 4) are meaningful to the mouse, then we should see a difference in behavioral responses to olfactory stimuli. To test this, we used a non-associative cross-habituation odor investigation paradigm (Figure 5A) in which investigation time was observed during a 50 sec presentation of a mineral oil vehicle followed by 4 successive presentations of HEX and then 1 presentation of HEPT at 5 min ITIs.

Both groups of mice investigated the mineral oil vehicle stimulus for comparable durations (Figure 5B; *t*_(28)_ = 0.216, *p* = 0.831) and also exhibited comparable investigation latencies (Figure 5C, compare open bars; *t*_(23)_ = 0.556, *p* = 0.584), suggesting that there were no inherent differences in locomotion or propensity to investigate novel stimuli. As shown in Figure 5B, there was also no change in average investigation time on the first odorant presentation compared to the mineral oil only trial (main effect of trial: *F*_(1, 28)_ = 0.002, *p* = 0.962, η_p_^2^ = 0.00), and no significant 2-way interaction of trial type and genotype (*F*_(1, 28)_ = 0.197, *p* = 0.661, η_p_^2^ = 0.007), demonstrating that OMP expression did not prevent the initial detection of a novel odorant. However, when we compared the latency to initiate stimulus investigation during these 2 trial types between groups we found an OMP expression (OMP^−/−^; OMP^+/+^) × trial type (mineral oil only; HEX_1_) interaction (Figure 5C; *F*_(1, 23)_ = 6.247, *p* = 0.020, η_p_^2^ = 0.214). OMP^+/+^ subjects exhibited a significant decrease in their latency to investigate the first presentation of HEX compared with their latency to investigate the mineral oil vehicle stimulus (within-subjects comparison, *t*_(13)_ = 2.484, *p* = 0.027), whereas OMP^−/−^ subjects exhibited no difference in their latency to investigate the mineral oil and HEX stimuli (within-subjects comparison, *t*_(10)_ = 1.189, *p* = 0.262). Moreover, the OMP^−/−^ group tended to take longer to begin investigating HEX during its first presentation than the OMP^+/+^ subjects (*t*_(23)_ = 2.065, *p* = 0.050). Thus, while OMP^−/−^ mice perceived the odor stimulus during its first presentation (Figure 5B), their behavioral responses were slightly delayed (Figure 5C).

To evaluate differences in habituation to HEX over successive presentations, total investigation time (sec) was analyzed via a 2-way mixed ANOVA, as described above. This analysis yielded a significant genotype × trial interaction (Figure 5B; *F*_(3, 84)_ = 5.702, *p* = 0.001, η_p_^2^ = 0.169). Specifically, OMP^+/+^ mice exhibited a significant reduction in the amount of time spent investigating HEX across 4 consecutive presentations (*post hoc* ANOVA, main effect of trial, *F*_(3, 54)_ = 36.267, *p*<0.001, η_p_^2^ = 0.668), whereas OMP^−/−^ mice did not exhibit a significant reduction in their investigation times (*post hoc* ANOVA, main effect of trial, *F*_(3, 30)_ = 0.87, *p* = 0.468, η_p_^2^ = 0.08), suggesting that only OMP^+/+^ mice fully habituated to the odor stimulus. We switched the odor stimulus to HEPT after the fourth habituation trial and observed a very modest and non-significant increase in the investigation time of the OMP^+/+^ group (Figure 5B; *t*_(18)_ = 1.846, *p* = 0.081), suggesting that, on average, cross-habituation occurred in these mice. Similarly, there was no change in average investigation time in the OMP^−/−^ group (Figure 5B; *t*_(10)_ = 0.183, *p* = 0.859). However, unlike the OMP^+/+^ group, it is unclear if this is indicative of cross-habitation because OMP^−/−^ mice did not exhibit complete behavioral habituation to HEX (Figure 5B).

## Discussion

In the present work we expanded on previous reports showing changes in OSN response dynamics, magnitudes, and odorant selectivity in OMP knockout mice [13], [14]. In our OMP-spH model that visualizes total odorant-evoked neurotransmitter release from OSNs *in vivo*, we found that odorant presentation evoked synaptic input to more olfactory bulb glomeruli in OMP^−/−^ mice than in OMP^+/−^ mice (Figure 1), and that this was at least partially due to a decrease in odorant selectivity across glomerular populations (Figure 2). Surprisingly, the total neurotransmitter release evoked by a 6 sec odorant presentation was found to be comparable between OMP heterozygous and knockout mice (Figure 3), though the responses in OMP^−/−^ were significantly smaller early in the odorant presentation (Figure 4). OMP^−/−^ mice were found to behave very differently than wild-type mice in an olfactory habituation task (Figure 5), where OMP^−/−^ mice exhibited a delayed onset in their initial investigation of a novel odor (although the total duration of this investigation was comparable between groups) and much less evidence of habituation across odorant presentations.

The decreased odorant selectivity of OSNs in OMP knockout mice is consistent with a previous report showing similar effects during patch-clamp experiments in single OSNs that express the MOR23 receptor [14]. The present findings demonstrate that OMP knockouts also exhibit reduced OSN odorant selectivity during natural odorant presentations *in vivo* and across OSN populations. It remains unclear whether this selectivity is conveyed by a direct action of OMP on the olfactory receptor or by some thresholding function that gates out nonspecific responses to suboptimal odorants prior to signal amplification in the OSN cilium.

Interestingly, OSNs in OMP knockout mice exhibited less total odorant-evoked neurotransmitter release (smaller spH signals) than those from heterozygous mice when assessed within the first two seconds of an odorant presentation (Figure 4) but “caught up” later in the odorant presentation such that the total neurotransmitter release was comparable towards the end of the trial (Figure 3). The delayed initial response is consistent with many reports from recordings of both single OSNs and populations of OSNs in the epithelium [13], [14], [21]. These reports indicated that the offset of the odorant-evoked response was delayed as well in OMP-null OSNs, potentially explaining how these OSNs could release more neurotransmitter than controls in the later part of the odorant presentation. Here we demonstrate that the primary odorant representations evoked in OMP^−/−^ mice indeed take longer to fully develop, but eventually become comparable in magnitude to those of mice that do express OMP. This delayed response may explain why OMP knockout mice can perform comparably to controls on olfactory guided tasks that provide plenty of time for odor sampling (Figure 5, first HEX trial; see also [15], [16]), though they may exhibit different patterns of errors [16]. It also explains why our earlier reports using the OMP-spH mouse line failed to observe differences in peak response magnitudes between homozygous and heterozygous mice [23].

Though it is difficult to causally link the changes in OSN neurophysiology to the differences in odorant-guided behavior, the delayed onset of initial odor investigation and the reduced behavioral habituation in OMP-null mice may reflect the underlying difference in the timing of OSN synaptic output, whereby the delayed synaptic output from OSNs results in a corresponding delay in investigative behavior and somehow impedes the normal process of habituation. We did not observe any differences between OMP-null and OMP-expressing mice in their ability to discriminate between two highly similar odorants; neither group showed dis-habituation from HEX to HEPT. However, it is difficult to interpret the behavior of OMP-null mice as being indicative of cross-habituation between the two odorants because these subjects showed little evidence of habituation to the first odorant.

Because the spH data provide an aggregate signal of neurotransmitter release from the population of OSNs innervating each glomerulus, we cannot in principle distinguish between increased exocytosis at each synapse and an increased total number of synapses in the population. In the present study, we found that total odorant-evoked neurotransmitter release was no different between OMP-null and OMP-heterozygous mice, despite a previous report that glomeruli in OMP-null mice include significantly more axodendritic synapses (which may arise from OSNs) than wild-type mice [32]. However, we cannot rule out the possibility that the OSNs in our OMP-null mice made more synapses than the OMP-expressing mice but compensated for this increase by somehow reducing their odorant-evoked neurotransmitter release per synapse. Similarly, we cannot entirely exclude the possibility that the slower response kinetics between our OMP-null and OMP-heterozygous mice somehow reflected an increased expression of spH in the OMP-null mice, which were homozygous for spH. However, previous experiments in olfactory bulb slices from homozygous spH mice showed an excellent correspondence between electrically-evoked presynaptic spH signals and postsynaptic currents in wild-type mice [24]. Highly synchronous, electrically-evoked neurotransmitter release can evoke a transient change in synaptic pH that partially masks the rise of the spH signal [28], but this transient occurs on the scale of milliseconds (not seconds) and has not been observed with odorant presentations *in vivo*. An actual change in OSN response kinetics thus seems the most parsimonious explanation of the present results.

Because of its high levels of selective expression, the OMP promoter is frequently used for gene-targeted expression of scientifically useful constructs, including fluorescent markers [33], physiological indicators like synaptopHluorin [23], and optogenetic stimulation tools like channelrhodopsin [34]. However, this application in gene-targeted models potentially disrupts the normal function of OMP in olfactory physiology. The present results indicate the need for caution in interpreting data from mice with altered OMP expression, particularly with regard to odorant selectivity and habituation assays.
